# The Relationship of Internet Gaming Addiction and Suicidal Ideation among Adolescents: The Mediating Role of Negative Emotion and the Moderating Role of Hope

**DOI:** 10.3390/ijerph20043375

**Published:** 2023-02-15

**Authors:** Yuntian Xie, Qian Yang, Fan Lei

**Affiliations:** Department of Applied Psychology, Changsha Normal University, Changsha 410100, China

**Keywords:** internet gaming addiction, suicidal ideation, negative emotion, hope

## Abstract

Recently, internet gaming addiction and suicide have been global public health issues among adolescents. This study used convenience sampling and surveyed 1906 Chinese adolescents to investigate the relationship between internet gaming addiction and suicidal ideation and the role of negative emotion and hope in the relationship between the two. The results showed that the detection rate of internet gaming addiction among adolescents was 17.16% and the detection rate of suicidal ideation was 16.37%. Moreover, there was a significant positive correlation between internet gaming addiction and suicidal ideation. Negative emotion partially mediated the relationship between internet gaming addiction and suicidal ideation. In addition, hope moderated the relationship between negative emotion and suicidal ideation. The effect of negative emotion on suicidal ideation decreased as hope increased. These findings suggest that the role of emotion and hope in coping with adolescent internet gaming addiction and suicidal ideation should be emphasized.

## 1. Introduction

Recently, suicide has become a public health problem on a global scale. Suicide includes suicidal ideation, attempted suicide, and death by suicide [1]. Suicidal ideation, defined as “thinking, contemplating, or planning suicide” [2], is considered an important risk factor for suicide [3]. It is the third leading cause of adolescent death [4]. According to a survey, 18–25% of Chinese adolescents have had suicidal ideation [5]. Behavioral problems and negative emotions have also been identified as important risk factors for suicidal ideation in adolescents [6,7,8].

### 1.1. The Relationship between Internet Gaming Addiction and Suicidal Ideation

Internet gaming addiction (IGA) is a common behavioral problem [9]. The persistent and repeated internet use for gaming causes significant harm or distress in a person’s life [10]. Its main characteristic is the excessive or compulsive use of internet-based games [11].

According to the stress-susceptibility model of suicide, suicide results from the interaction of stressors and susceptibility factors, with severe feelings of dependence as a stressor [12]. A meta-analysis study that included 25 studies found that suicidal ideation was significantly higher in internet addicts than non-internet addicts [13]. A more recent meta-analysis that included 12 studies found a link between problematic play and suicidal ideation [2]. Social learning theory suggests that suicide is a learned problem-solving style [14]. Online gamers may be more likely to be exposed to suicidal risk factors in video games [15], which may lead to suicidal ideation. The current study concluded that adolescent internet gaming addiction is closely related to suicidal ideation.

### 1.2. The Mediating Role of Negative Emotion

One of the risk factors that has received the most attention in suicidal ideation research is negative emotion [16]. In addition, negative emotions are an individual’s subjective unpleasant or painful emotional experiences [16]. According to the interpersonal theory of suicide, poor interpersonal relationships can increase an individual’s negative emotions, leading to suicidal ideation [17]. Moreover, according to the escape theory of suicide, the purpose of suicide is to escape from unbearable emotions or thoughts [18]. Suicide is usually a maladaptive response to uncontrollable and extremely painful negative emotional problems [19]. Therefore, suicidal ideation may arise due to strong negative emotions [16]. The results of a cross-lagged analysis showed that negative emotions were predictive of suicidal ideation in adolescents [20].

Negative emotions are linked not only to suicidal ideation but also to internet gaming addiction. Studies have found that addicts are vulnerable to emotional instability because they have been disconnected from real life for a long time [21]. Moreover, internet gaming addiction is a negative coping style that can further aggravate the symptoms of negative emotions [22,23]. The interpersonal theory of suicide suggests that an individual’s unmet sense of belonging is a major factor in suicide [17,24]. The central assumption of the theory is that suicidal thoughts arise when perceived burdens and frustrating feelings of belonging are high [25]. Adolescents addicted to the internet have poorer quality relationships and more homogeneous coping strategies and are more likely to experience negative emotions such as anxiety and depression [26]. Therefore, this present study suggests that negative emotions are likely to mediate between adolescent internet gaming addiction and suicidal ideation. 

### 1.3. The Moderating Effect of Hope

Snyder’s hope theory states that hope is a thinking process comprised of two basic components: motivational thinking and path thinking [27,28]. The former requires goal identification, while the latter necessitates goal planning. It has been suggested that a factor that may confer protection against the development of suicidal ideation and behaviour is hope, a future orientated construct that features [29]. Several studies have found that hope reduces the effect of psychological stress on suicidal ideation [30,31]. Kwok and Gu (2019) found that hope moderates the relationship between depressive symptoms and suicidal ideation in adolescents and that the effect of depressive symptoms on suicidal ideation was significantly reduced in individuals with high hope compared to those with low hope [32].

Furthermore, positive self-evaluations, according to the Schematic Appraisals Model of Suicide, have a buffering effect on suicidal thoughts and behaviors [1]. This positive cognitive model of hope can assist individuals in dealing positively with difficulties and obstacles in their development [28]. Based on this, the present study suggests that hope may influence the role of negative emotion.

### 1.4. The Present Study

**Figure 1:**
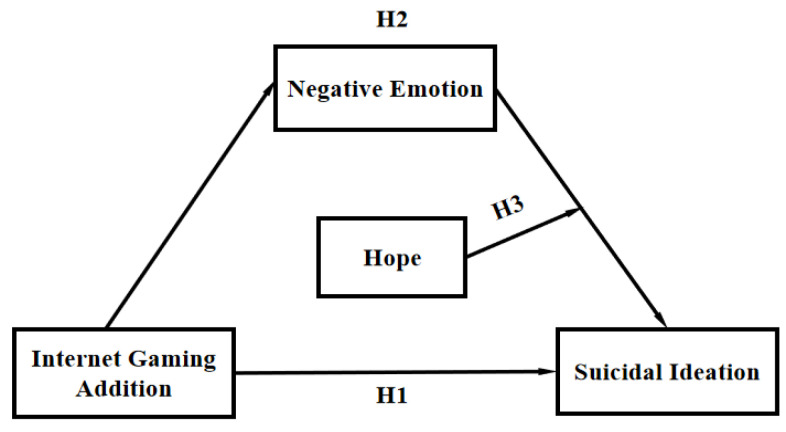
The conceptual model.

In order to explore the relationship between internet gaming addiction and suicidal ideation in-depth, a mediating role model with regulation was developed in this study (see Figure 1). This model investigated the direct relationship between internet gaming addiction and suicidal ideation and the mediating and moderating roles of negative emotion and hope. Our hypotheses were as follows.

**Hypothesis 1. (H1)**: *Internet gaming addiction might be positively associated with suicidal ideation.*

**Hypothesis 2. (H2)**: *Negative emotions might mediate the relationship between internet gaming addiction and suicidal ideation.*

**Hypothesis 3. (H3)**: *Hope might moderate the mediating role of negative emotion in the relationship between internet gaming addiction and suicidal ideation. Specifically, it will moderate the back-end path of the mediating model.*

## 2. Method

### 2.1. Participants

Using convenience sampling, 1979 questionnaires were distributed to four middle schools in Ganzhou, China, and 1906 valid questionnaires (96.31%) were returned. There were 914 boys and 974 girls, with 18 missing; 650 were in first grade, 587 in second grade, and 664 in third grade, with five missing. Furthermore, there were 978 students from urban areas and 916 students from rural areas, with 12 missing. The mean age was 14.09 (*SD* = 1.01), with an age range of 12 to 17 years.

### 2.2. Procedures

This study was carried out in groups as a class. The questionnaire was answered anonymously. After obtaining the consent of the students, a uniform instruction was read out by the main examiner, and the students were asked to complete it within a specified time. After completion, the questionnaires were collected on the spot. 

### 2.3. Measures

#### 2.3.1. Internet Gaming Addition

The Internet Gaming Addiction Questionnaire was used, as developed by Yu et al. [33]. The questionnaire contains 11 items and 1 factor. A three-point Likert scale is used, with scores of 0 for “never,” 0.5 for “sometimes,” and 1 for “often.” The higher the score, the greater the tendency to internet gaming addiction. If the total score … 5, it is regarded as internet gaming addiction. In this study, the scale’s alpha coefficient was 0.859.

#### 2.3.2. Suicidal Ideation

The Self-rating Idea of the Suicide Scale developed by Xia et al. [34] was used. The scale contains 26 items and 4 factors: despair, optimism, sleep, and masking. Among them, the masking dimension reflects the extent to which the test was masked by the subjects. If the masking factor score is low, then it indicates that the data is a good reflection of the individual’s true state of affairs. The scale is scored using a “yes” or “no” response. “Yes” is scored as 1, and “No” is scored as 0. If the total score of despair, optimism, and sleep was …12, and the score of the masking dimension was <4, it is considered suicidal ideation. In this study, the alpha coefficients for the four factors ranged between 0.575 and 0841.

#### 2.3.3. Negative Emotion

The Negative Affect subscale of the Positive and Negative Affect Schedule developed by Watson [35] and translated by Zhang et al. [36] was used. The scale contains 10 items and 1 factor. A five-point Likert scale is used, with 1 representing “nearly none” and 5 representing “extremely many.” The higher the score, the greater the degree of negative emotion. In this study, the scale’s alpha coefficient was 0.858.

#### 2.3.4. Hope

The Children’s Hope Scale developed by Snyder et al. [37] and translated by Zhao and Sun [38] was used. The scale consists of 6 items and 2 factors: path thinking and motivational thinking. A six-point Likert scale is employed, with 1 representing “never” and 6 representing “always”. The higher the score, the greater the level of hope. In this study, the alpha coefficients for the two factors were 0.799 and 0.818, respectively. 

### 2.4. Statistical Analysis

SPSS 25.0 was used for frequency analysis, descriptive statistics, *t*-tests and correlation analysis. The PROCESS 4.0 macro program plug-in developed by Hayes [39] was used to perform the mediating effect test and the mediating effect test with moderation. Based on the current test models provided by PROCESS, Model 4 was first selected to test the simple mediation model and then Model 14 was selected to test the mediation model with moderation (the moderating variable moderates the back-end path of the mediation model) in this study. Furthermore, the study examined the trajectory of the simple slope using the Johnson-Neyman methods to explain better the continuum of moderating effects. Data were tested for significance of effect using the bias-corrected percentile bootstrap method, with 5000 replicate samples and 95% confidence intervals calculated. Finally, because the data for this study were obtained from the subjects’ self-reports, there may be common method bias. Harman’s one-factor analysis was used to test for common method bias. It was found that there were 10 factors with eigenvalues greater than one, and the first factor explained 19.48% of the variance, which was less than 40%. Therefore, there was no significant common method bias in this study. In addition, violin plots were drawn using JASP 0.16.1.

## 3. Results

### 3.1. Frequency Analysis, Descriptive Statistics, t-Tests and Correlation Analysis

**Figure 2:**
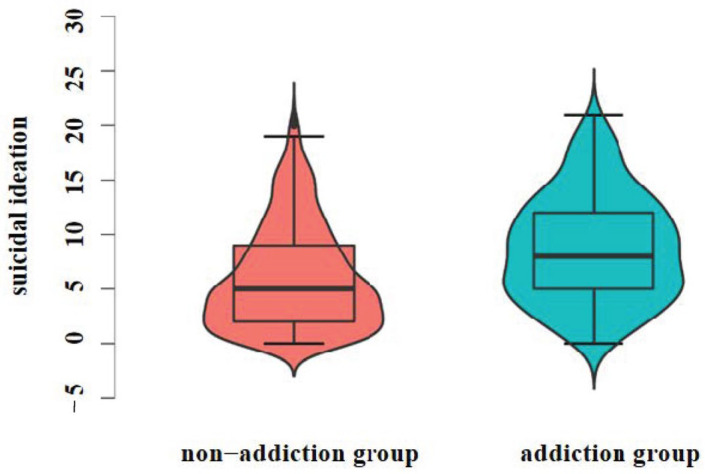
Differences in suicidal ideation between the non-addiction group and addiction group.

The study found that 327 (17.16%) junior high school students had an internet gaming addiction score greater than or equal to 5. A total of 312 students (16.37%) scored greater than or equal to 12 on suicidal ideation and less than 4 on masking. Suicidal ideation was significantly higher in the internet gaming addiction group (8.77 ± 4.90) than in the non-addiction group (6.02 ± 4.75), *t* = 9.51, *p* < 0.001, Cohens’ *d* = 0.58. Suicidal ideation scores in the non-addiction group were mostly below 5, as shown in Figure 2, whereas suicidal ideation scores in the addiction group were mainly concentrated between 5 and 10 points. 

The results of the test for differences in the main variables based on gender and place of residence (see Table 1) revealed that middle school boys had significantly higher levels of internet gaming addiction and hope than girls (*p* < 0.001). Suicidal ideation and negative emotion were significantly higher in junior high school girls than boys (*p* < 0.001). Similarly, rural students had significantly higher levels of internet gaming addiction, suicidal ideation, and negative emotion than urban students (*p* < 0.01). Rural students had significantly lower levels of negative emotion and hope than urban students (*p* < 0.001).

The correlation analysis revealed (see Table 2) that there was a significant positive correlation between age and internet gaming addiction and a significant negative correlation between age and hope. Internet gaming addiction had a significant positive correlation with suicidal ideation and negative emotion (*p* < 0.001). Similarly, suicidal ideation showed a significant positive correlation with negative emotion (*p* < 0.001). Hope was found to have a significant negative correlation with internet gaming addiction, suicidal ideation, and negative emotion (*p* < 0.001).

### 3.2. Test for Mediating Effects with Moderation

First, the study tested the mediating role of negative emotion. The results showed that after controlling for the effects of age, gender (0 = girls, 1 = boys), and place of residence (0 = rural, 1 = urban), internet gaming addiction significantly and positively predicted suicidal ideation (*c* = 0.38, *p* < 0.001, see Equation (1) in Table 3). Moreover, internet gaming addiction significantly predicted negative emotions (*a* = 0.06, *p* < 0.001, see Equation (2) in Table 3). And when both internet gaming addiction and negative emotion were included in the regression equation, negative emotion significantly and positively predicted suicidal ideation (*b* = 3.00, *p* < 0.001). Likewise, internet gaming addiction still significantly and positively predicted suicidal ideation (*c*′ = 0.19, *p* < 0.0001). The results of the mediating effect test showed a significant mediating effect of negative emotion between internet gaming addiction and suicidal ideation, *ab* = 0.19, Boot *SE* = 0.02, Boot 95% CI = [0.16, 0.22].

Then, the study tested the moderating effect of hope on the back-end path of the mediation model. The results showed that the interaction between negative emotion and hope had a significant effect on suicidal ideation (*B* = −0.22, *p* < 0.05, see Equation (3) in Table 3). Additionally, as shown in Table 4, the mediating effect of negative emotion diminished at three different levels of hope (from low to high). These findings suggested hope moderated the mediating effect of negative emotions between internet gaming addiction and suicidal ideation, indicating that a moderated mediation model existed between internet gaming addiction, suicidal ideation, negative emotion, and hope.

**Figure 3:**
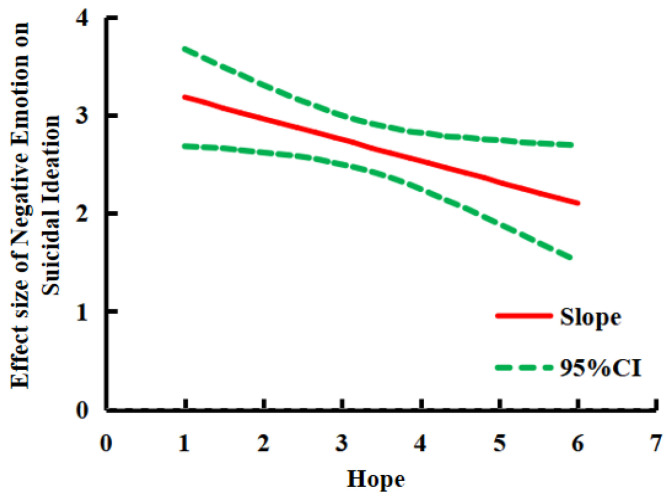
The trajectory of simple slope change.

Furthermore, in examining the trajectory of the simple slope using the Johnson-Neyman method, the results showed that the effect of negative emotions on suicidal ideation continued to decline along with the increase in the level of hope (see Figure 3). It suggested that hope can play a buffering role in the process of negative emotions affecting suicidal ideation.

## 4. Discussion

### 4.1. The Findings of the Current Study

This present study found that internet gaming addiction was significantly and positively associated with suicidal ideation. This result supports hypothesis 1 and is consistent with previous research findings [6,7]. Adolescents addicted to online games tend to depersonalize, which affects their normal social functioning and makes it difficult for them to satisfy their sense of belonging, leading to suicidal ideation [24].

Secondly, this study found that negative emotions could mediate the relationship between internet gaming addiction and suicidal ideation. This result verifies hypothesis 2. Adolescents are in a stage of rapid physical and mental development. If they are addicted to online games, they are prone to negative emotions and are emotionally unstable, generating extreme thoughts of, for example, wanting to end their lives. In the present study, internet gaming addiction significantly and positively predicted negative emotions. This is consistent with previous research findings [21,22]. Individuals who were addicted to online games were prone to negative emotions. Furthermore, suicidal ideation was significantly predicted by negative emotions. This result is similar to the previous findings [16,20]. If an individual cannot tolerate such painful negative emotions, he/she may have extreme thoughts such as suicide.

Finally, this study also found that hope significantly moderated the mediating role of negative emotions. This result verifies hypothesis 3. Hope is a protective factor [40]. Adolescents with high hope had a significantly lower negative mediating role between internet gaming addiction and suicidal ideation than those with low hope. Moreover, suicidal ideation was less influenced by negative emotions in high-hopeful adolescents than in low-hopeful adolescents.

### 4.2. Theoretical Contributions

In this study, we developed a moderated mediating role model to investigate the relationship between adolescent internet gaming addiction, suicidal ideation, negative emotions, and hope. 

First, we observed the mediating role of negative emotions. This finding supports both the avoidance theory of suicide and the interpersonal theory of suicide. According to the former theory, individuals have an increased probability of suicidal ideation following contemplation of failure, particularly when combined with negative emotions [41]. According to the latter theory, negative emotions such as loneliness and frustration of belonging are a manifestation of interpersonal dysfunction [42]. These emotions are also prone to suicidal ideation if they are present in the individual for a long time.

In addition, we verified the value of hope. Its significance is primarily reflected in its protective function. Although some studies have shown that hope has a protective function [29,30,31], this study enriches the existing research on the topic of hope by considering it in the context of the relationship between internet gaming addiction, suicidal ideation, and negative emotions.

### 4.3. Practical Implications

This study revealed the mechanism of adolescent suicidal ideation generation, which helps people better understand the risk factors and protective factors of suicidal ideation. 

First, we should consider the impact of internet gaming addiction on adolescent suicidal ideation. In the process of preventing adolescents from developing suicidal ideation, we should pay closer attention to those adolescents who are addicted to online games. If we can teach them how to evaluate online games and deal with internet gaming addiction properly, they will be more likely to re-examine life and re-understand the meaning and value of life, and they will be less likely to develop suicidal thoughts. 

Secondly, we should consider the influence of negative emotions on adolescents’ suicidal thoughts. More thematic activities about the emotional experience and emotional catharsis should be included in youth education activities. We should guide adolescents through these activities to master the methods of adjusting their emotions, coping with negative emotions correctly, and enriching positive emotional experiences. 

Finally, we should pay attention to the protective role of hope. One concern is that this study found a positive correlation between age and online game addiction and a negative correlation with hope. While the generalization of this result needs to be treated with caution, it needs to provoke thought. It is possible that factors such as adolescent rebellion, academic pressure, and family functioning may have contributed to this phenomenon. We should focus on guiding adolescents’ aspirations and expectations for the future and raising their level of hope. Goals are the core of hope theory [43], and values are desirable aims or basic principles of an individual’s life [44]. To this end, we should guide our young people to establish the right values, find their goals in life, and continually strengthen their sense of hope.

### 4.4. Limitations and Directions for Future Research

There are also some limitations in this study. Firstly, this study relied on self-reported questionnaire data. Future studies can obtain richer data through observation, interviews, and questionnaire data collection. Second, this study is a cross-sectional study, and it is difficult to reflect on the causal relationship between internet gaming addiction and suicidal ideation. Future research can investigate the relationship between the two using a cross-lagged research design. Third, the sample of this study was only junior high school students in one region of China. Future studies could include adolescents from different regions or cultural backgrounds to confirm the findings of this study. Future studies could focus on and compare the differential performance of adolescents from different cultural backgrounds (e.g., Eastern and Western cultures, collectivist and individualist cultures) on factors such as internet gaming addiction and suicidal ideation.

## 5. Conclusions

The detection rates of both internet gaming addiction and suicidal ideation among adolescents were close to 20%. This problem must be taken very seriously. Moreover, internet gaming addiction and suicidal ideation in adolescents are closely related. Suicidal ideation may be influenced by internet gaming addiction both directly and indirectly through negative emotions. In addition, hope may moderate the mediating role of negative emotion in the relationship between Internet gaming addiction and suicidal ideation. Specifically, as hope increases, the role of negative emotion as a mediator in the relationship between the two decreases. As a result, the role of emotion and hope in coping with adolescent internet gaming addiction and suicidal ideation should be emphasized.

## Figures and Tables

**Table 1:** Differences in the main variables by gender and residence (*M ± SD*).

	Gender				Place of Residence			
	Boys (*n* = 914)	Girls (*n* = 974)	*t*	Cohens’ *d*	Urban (*n* = 978)	Rural (*n* = 916)	*t*	Cohens’ *d*
1. IGA	3.06 ± 2.20	1.92 ± 1.97	11.90 ***	0.55	2.23 ± 2.12	2.73 ± 2.18	−5.08 ***	−0.23
2. SI	5.99 ± 4.66	6.95 ± 5.05	−4.29 ***	−0.20	6.10 ± 4.75	6.90 ± 5.00	−3.63 ***	−0.17
3. NE	2.01 ± 0.77	2.17 ± 0.76	−4.56 ***	−0.21	2.05 ± 0.77	2.14 ± 0.77	−2.74 **	−0.13
4. Hope	3.53 ± 1.15	3.31 ± 1.04	4.31 ***	0.20	3.63 ± 1.13	3.19 ± 1.01	8.77 ***	0.40

Note. IGA = internet gaming addiction, SI = suicidal ideation, NE = negative emotion; *** *p* < 0.001, ** *p* < 0.01.

**Table 2:** Descriptive statistics results and correlation coefficients.

	*M*	*SD*	1	2	3	4
1. Age	14.09	1.01	-			
2. IGA	2.47	2.17	0.07 **	-		
3. SI	6.49	4.88	0.03	0.29 ***	-	
4. NE	2.09	0.77	0.03	0.30 ***	0.53 ***	-
5. Hope	3.42	1.10	−0.11 ***	−0.16 ***	−0.41 ***	−0.22 ***

*** *p* < 0.001, ** *p* < 0.01.

**Table 3:** Tests for mediating effects with moderation.

	Equation (1) (Dependent Variable: SI)			Equation (2) (Dependent Variable: NE)			Equation (3) (Dependent Variable: SI)		
	*B*	*t*	95% CI	*B*	*t*	95% CI	*B*	*t*	95% CI
Age	0.02	0.17	[−0.19, 0.23]	0.01	0.25	[−0.03, 0.04]	−0.11	−1.24	[−0.28, 0.06]
Boys	−1.83	−8.23 ***	[−2.26, −1.39]	−0.31	−8.82 ***	[−0.38, −0.24]	−0.59	−3.12 ***	[−0.96, −0.22]
Urban	−0.36	−1.63	[−0.78, 0.07]	−0.01	−0.36	[−0.08, 0.05]	0.11	0.60	[−0.25, 0.47]
IGA	0.38	14.61 ***	[0.33, 0.43]	0.06	15.36 ***	[0.05, 0.07]	0.16	6.81 ***	[0.11, 0.21]
NE							3.39	9.88 ***	[2.61, 4.20]
Hope							−0.89	−4.15 ***	[−1.33, −0.44]
NE × Hope							−0.22	−2.17 *	[−0.41, −0.02]
*R^2^*	0.12			0.13			0.39		
*F*	62.14 ***			66.80 ***			171.47 ***		

*** *p* < 0.001, * *p* < 0.05.

**Table 4:** The mediating effect of negative emotion at different levels of hope.

	Hope	Effect	Boot SE	Boot CI
The mediating effect of negative emotion	*M* − 1*SD*	0.18	0.02	[0.15, 0.21]
	*M*	0.17	0.01	[0.14, 0.19]
	*M* + 1*SD*	0.15	0.02	[0.12, 0.18]

## Data Availability

The data presented in this study are available upon request from the corresponding author.
